# Smell and taste in idiopathic blepharospasm

**DOI:** 10.1007/s00702-021-02366-4

**Published:** 2021-06-28

**Authors:** Julie Gamain, Thorsten Herr, Robert Fleischmann, Andrea Stenner, Marcus Vollmer, Carsten Willert, Birgitt Veit, Bernhard Lehnert, Jan-Uwe Mueller, Frank Steigerwald, Frank Tost, Martin Kronenbuerger

**Affiliations:** 1 Department of Neurology, University of Greifswald, Greifswald, Germany; 2 Department of Neurology, Paracelsus Clinic Zwickau, Zwickau, Germany; 3 Institute of Bioinformatics, University of Greifswald, Greifswald, Germany; 4 Neurology Group Practice, Stralsund, Germany; 5 Neurology Group Practice, Neubrandenburg, Germany; 6 Department of Otorhinolaryngology, University of Greifswald, Greifswald, Germany; 7 Department of Neurosurgery, University of Greifswald, Greifswald, Germany; 8 Department of Ophthalmology, University of Greifswald, Greifswald, Germany; 9 Department of Neurology, Johns Hopkins University, Baltimore, MD USA; 10 Department of Neurology, Medical School OWL, University of Bielefeld, Bielefeld, Germany

**Keywords:** Non-motor deficit, Network, Basal ganglia, Thalamus, Cerebellum, Cortex

## Abstract

The pathophysiology of blepharospasm is incompletely understood. Current concepts suggest that blepharospasm is a network disorder, involving basal ganglia, thalamus, cortex, and, possibly, the cerebellum. Tracing, imaging, and clinical studies revealed that these structures are also concerned with olfaction and taste. Because of this anatomical overlap, dysfunction of the chemical senses in blepharospasm is expected. Injections of botulinum toxin into the eyelid muscles are the first-line treatment of blepharospasm. Yet, the effects of botulinum toxin on the chemical senses have not been systematically assessed. To contribute to a better understanding of blepharospasm, olfactory and gustatory abilities were assessed in 17 subjects with blepharospasm and 17 age-/sex-matched healthy controls. Sniffin Sticks were used to assess odor threshold, odor discrimination, and odor identification. Results of these three Sniffin Sticks subtests were added to the composite olfactory score. The Taste Strips were applied to assess taste. In an adjacent study, we assessed the sense of smell and taste in eight subjects with blepharospasm before and 4 weeks after botulinum toxin treatment. Subjects with blepharospasm had significantly lower (= worse) scores for odor threshold and for the composite olfactory score than healthy controls, while odor discrimination, odor identification, and the composite taste score were not different between groups. The adjacent study revealed that botulinum toxin did not impact the chemical senses. In this study, subjects with blepharospasm had a lower (= worse) odor threshold than healthy controls. As olfaction is important in daily life, findings justify further research of olfaction in blepharospasm.

**Supplementary Information:**

The online version contains supplementary material available at 10.1007/s00702-021-02366-4.

## Introduction

Blepharospasm (BSP) is a dystonia subtype that is characterized by stereotyped, bilateral, and synchronous spasms of the orbicularis oculi muscle in combination with sensory tricks and increased blinking (Defazio et al. 2017). Subjects with BSP may be functionally blind and unable to live a normal life due to spasms of the eyelid muscles (Valls-Sole and Defazio 2016). Additionally, sensory (such as dry eye and photophobia), psychiatric (such as depression and anxiety), and cognitive alterations (such as memory and executive function) are possible or putative non-motor deficits of BSP which negatively impact the quality of life (Ferrazzano et al. 2019; Girach et al. 2019). BSP may present secondary to a different illness, such as focal lesion in the brain, or in combination with other dystonias such as oro-mandibular dystonia (Albanese et al. 2013). When BSP occurs in isolation and is unrelated to a different condition, it is termed idiopathic BSP (Albanese et al. 2013). Although being the most common cranial dystonia and the second most common focal dystonia (ESDE 2000; Defazio et al. 2017), the pathophysiology of idiopathic BSP is incompletely understood (Defazio et al. 2017). A better understanding of its pathophysiology may provide diagnostic biomarkers or novel gateways for its treatment. Such understanding is highly relevant as motor and non-motor alterations cause significant disability for subjects with idiopathic BSP (Ferrazzano et al. 2019; Girach et al. 2019).

Current concepts on the pathophysiology of idiopathic BSP suggest that it is related to alterations in a network of sensorimotor control that includes the sensorimotor and parietal cortex, basal ganglia, thalamus, brainstem, and, possibly, the cerebellum (Defazio et al. 2017; Chirumamilla et al. 2019; Glickman et al. 2020; Mascia et al. 2020). Tracing studies in animals (Ikai et al. 1992; Price 2003), functional imaging studies in healthy human subjects (Sobel et al. 1998; Savic et al. 2000) and clinicopathological studies (Mainland et al. 2005; Zobel et al. 2010; Wistehube et al. 2018) revealed that the basal ganglia, thalamus, cerebellum, and sensorimotor cortex are also concerned with the processing of information related to the chemical senses. Because of the anatomical overlap of structures involved in the pathophysiology of idiopathic BSP and the chemical senses, an impairment of the sense of smell and taste may be found in subjects with idiopathic BSP. This idea is supported by diminished chemical senses in subjects with cervical dystonia (CD) (Marek et al. 2018; Herr et al. 2020), where alterations in the sensorimotor control network are also found (Jinnah et al. 2013, 2017). Although largely overlooked, the chemical senses fulfill fundamental aspects important for daily life (Croy et al. 2014; Doty 2019). For instance, they contribute to the quality of life, they can warn us of hazards, and they are important for social relations (Stevenson 2010; Gelstein et al. 2011; Croy et al. 2014). Diminished chemical senses may worsen the situation of subjects with idiopathic BSP. To contribute to a better understanding of idiopathic BSP, the sense of smell and the sense of taste were systematically assessed in subjects with idiopathic BSP and compared to matched healthy controls.

The pathophysiology of BSP is incompletely understood and there is no curative treatment for BSP at present (Hallett et al. 2008; Defazio et al. 2017). Thus, currently available treatment options for BSP are symptomatic (Hallett et al. 2008; Defazio et al. 2017). Injections of botulinum toxin (BoNT) into the eyelid muscles are the first-line treatment for subjects with idiopathic BSP (Hallett et al. 2013; Hassell and Charles 2020). BoNT blocks the release of acetylcholine from axon endings at the neuromuscular junctions to cause a flaccid paralysis (Figgitt and Noble 2002; Hallett et al. 2013; Hassell and Charles 2020). Yet, it may also diffuse to other structures such as the glands in the nose and mouth, where acetylcholine is also a neurotransmitter (Schulte-Mattler 2008; Hallett et al. 2013). As a result, BoNT treatment may diminish the functioning of these glands and cause dry mouth and dry nose (Hallett et al. 2013; Hassell and Charles 2020). As saliva in the mouth and mucus in the nose are important for olfactory and gustatory molecules to dissolve, to bind as well as to stimulate the olfactory and gustatory receptors (Rawal et al. 2016; Doty 2019), a decline in the sense of smell and taste may occur with BoNT treatment due to its side effects of dry mucous membranes (Hassell and Charles 2020). To the best of our knowledge, the effects of BoNT on olfactory and gustatory abilities have not thus far been systematically assessed. Therefore, the chemical senses in subjects with idiopathic BSP were assessed before and 4 weeks after BoNT injections in an adjacent study. Findings of this adjacent study were compared to healthy subjects, who were also assessed twice 4 weeks apart but without BoNT treatment.

## Methods

### Study subjects

Study participants were recruited through the Ophthalmology Department and Neurology Department at the University Hospital Greifswald before January 2020. Inclusion criteria for BSP subjects were standardized criteria for idiopathic BSP without evidence of a different neurological illness and/or other causes of involuntary lid closure (Defazio and Livrea 2004; Defazio et al. 2017). Healthy subjects matched for age, sex, education, and handedness of the BSP subjects were included as healthy controls. The exclusion criteria were chosen, so that no other neurological disease and no disorder of the peripheral olfactory and gustatory system could influence the test results. Exclusion criteria applied to both groups were a score below 26 in the Montreal Cognitive Assessment (MoCA) (Freitas et al. 2012), post-infectious olfactory dysfunction, post-traumatic olfactory dysfunction, olfactory dysfunction secondary to sinonasal disease, congenital olfactory dysfunction, idiopathic olfactory dysfunction, any history of or current radiation and chemotherapy, past or present tobacco smoking, use of central nervous system (CNS) active medication, anatomical deformities or pathologies of the mouth, ear and nose, medical or surgical conditions which could impede smell or taste, as well as history of head trauma, abnormal findings on routine neuroimaging studies and abnormal findings on laboratory work-up done for routine care (Jankovic and Orman 1987; Heckmann et al. 2003; Doty 2019). Also, excluded from the study were subjects taking medication or who were under exposure to toxins which cause dysfunction of the chemical senses (Hummel et al. 2016; Doty 2019; Kronenbürger and Pilgramm 2020).

After approval of the study protocol by the local ethics board, all subjects with the diagnosis of blepharospasm who presented to the Department of Ophthalmology and to the Department of Neurology outpatient clinics between January 2015 and January 2020 were identified in the hospital electronic record system of the University Hospital Greifswald. Thereafter, using the available health information in the hospital electronic medical records system, the inclusion and exclusion criteria were checked in the identified subjects with blepharospasms. In the following step, the remaining subjects with blepharospasms who met the inclusion criteria and did not have any exclusion criteria were mailed a letter about the study and*—if they were interested to voluntarily participate in the study—*invited to contact the study investigators. Subjects with BSP who showed interest and who contacted the study investigators were interviewed over the phone if they met the inclusion criteria and if they had no exclusion criteria. Thereafter, respective BSP subjects obtained an appointment for the study, where they were examined and once more interviewed in regards to inclusion and exclusion criteria. Subjects with BSP who met the inclusion criteria and did not have any exclusion criteria were considered to have idiopathic BSP.

Healthy controls were recruited by flyers, which were distributed at the Department of Ophthalmology and at the Department of Neurology outpatient clinics. As with the subjects with idiopathic BSP, healthy controls interested to participate in the study and who called the study investigators were interviewed over the phone and scheduled for an appointment for the study if they met the inclusion criteria and if they did not have any exclusion criteria. Other selection criteria were that the healthy controls matched age, sex, education, and handedness of the subjects with BSP. At the study appointment, healthy controls were examined and again interviewed regarding the inclusion and exclusion criteria.

As we wanted to assess olfactory abilities in a cohort of subjects with idiopathic BSP, subjective olfactory decline was neither an inclusion nor an exclusion criterion for subjects with BSP or healthy controls. Thus, for screening and subsequent recruitment for this study, study participants were not inquired about their subjective olfactory functioning before entering the study. Once the study participants were included in the study, they were asked about their subjective rating of their olfactory abilities and their gustatory abilities (please see “Clinical interview, exam, and scores”).

### Clinical interview, exam, and scores

In addition to clinical chart review, demographic as well as clinical data were collected during the in-person interview. A neurological exam in all study subjects was performed by a fellowship-trained, senior movement disorders neurologist (MK). The Jankovic Ratings Scale (JRS) (Jankovic and Orman 1987) and the Blepharospasm Severity Rating Scale (BSRS) (Defazio et al. 2015) were applied to assess BSP. To assess cognitive and psychiatric alterations that could potentially impede the chemical senses (Hedner et al. 2010; Kamath et al. 2018), the protocol included the MoCA (Freitas et al. 2012), the Trail-Making-Test (Brown et al. 1958), the Digit-Span-Test (de Paula et al. 2016), the FAS-Test (Machado et al. 2009), and the Brief Symptom Inventory (Franke 2000). All participants were examined in a well-rested state. An anterior rhinoscopy, as well as a clinical exam of the oral cavity were performed on all participants to exclude any conditions which could cause impairment of the chemical senses. BSP subjects on BoNT treatment were assessed three months after their last treatment when the effects of BoNT had wasted. To assess saliva production, the study participants were asked to place two cotton swabs in their check pouch for 1 min. The weight of the cotton swabs was measured before and after the subjects had them in their mouth.

All study participants were asked to gauge their overall olfactory ability (= subjective olfactory ability rating) and their overall gustatory ability (= subjective gustatory rating) on a scale from 0 (absent) to 10 (superb) before the beginning of the Sniffin Sticks and Taste Stripe test as described below.

An adjacent study assessed the effects of BoNT treatment on the chemical senses. BSP subjects currently treated with BoNT were either examined right before their next BoNT injections and then examined 4 weeks thereafter, or they were examined in reverse order. The BoNT treatment was applied consistent with standard injection protocols, in standard doses with four-to-six injection sites per eye (Hassell and Charles 2020). To document the effects of BoNT treatment, the JRS was done right before the BoNT injections and 4 weeks thereafter. Self-assessment of the effects of BoNT in the BSP subjects was done using the Global Assessment Score (Wabbels et al. 2011). Using this scale, the BSP subjects were asked to rate the response to BoNT treatment from − 4 to + 4 (“− 4” indicated marked worsening of symptoms, “0” no change, and “+ 4” marked improvement of symptoms). For comparison, matched healthy controls were assessed twice, 4 weeks apart but without BoNT treatment.

### Olfactory testing

The participants' sense of smell was assessed using Sniffin Sticks (Hummel et al. 1997). These included three subtests for odor threshold, odor identification, and odor discrimination. The Sniffin Sticks have the shape of a pen and a removable cap. The participants were requested not to eat or use chewing gum an hour before the examination. Taking sips of water was allowed for the participants. To prevent visual identification, all study participants were tested blindfolded. For olfactory testing, the cap of the Sniffin Sticks pen was removed and the pen was presented under the nostrils for 3 s. Sufficient breaks were made between the presentation of the pens.

To assess the odor threshold, 16 triplets were used. Each triplet consisted of one pen, which contained the odorant n-butanol in different dilutions, and two pens which contained an odorless solvent. Thus, the odor threshold test of the Sniffin Sticks had 16 sticks, which contained n-butanol. The Sniffin Sticks pen with the highest concentration of the odorant had an n-butanol concentration of 4%. The difference between the n-butanol dilution in the different pens which contained the odorant was stepwise with a ratio of 1:2 per step. At the beginning of the test, the study participants were offered the pen with the highest concentration of the odorant, so that they could familiarize themselves with the fragrance. Thereafter, the triplet with the lowest odor concentration was presented. The three pens of a triplet were presented in alternating order. In a forced-choice procedure (AFC-procedure), the subjects were instructed to decide in which pen they had perceived a fragrance. In case the study participants were uncertain, they had to guess the answer. If the participants gave an incorrect answer, the triplet with the next higher concentration was presented until the study participants gave the correct answer. If the participants gave the correct answer during the first attempt, the triplet with the same concentration was offered to them again. When subjects had again chosen the correct pen, the test continued with the next lower concentration. This was done until the participants gave an incorrect answer, and then, the concentration was increased. The change between increasing and decreasing concentration (or vice versa) resulted in turning points. Results were documented on a documentation sheet. A total of seven turning points were determined, with the last four being considered for evaluation. The mean was calculated from these four turning points. This mean value indicated the olfactory threshold concentration of the study participants and could take values between zero (absent) and 16 (excellent). Thus, a low number in the odor threshold test also called “low odor threshold” indicated a worse or an impaired odor threshold. For example, if the group of subjects with idiopathic BSP had a lower odor threshold than the group of healthy controls, the odor threshold of subjects with BSP was deemed worse than the odor threshold of healthy controls.

For the assessment of odor discrimination, 16 triplets were used. A triplet included two pens with the same odor and one pen with a different odorant. The participants had to identify the stick with the different fragrances during this examination. The participants could achieve a maximum of 16 points. A lower score in the odor discrimination test indicated a worse performance than did a higher score.

Odor identification was assessed with 16 pens. These had different fragrances that represented known smells; for example, orange, leather, coffee, or garlic. After one pen was presented, four predetermined answer options were read out to the participants and they had to indicate which of the four was most likely the correct answer. The correct answers were noted and added at the end of the examination. Similar to the other tests, a maximum total of 16 points was possible. A lower score in the odor identification test indicated a worse performance than did a higher score.

The three Sniffin Sticks subtests (odor threshold, odor discrimination, and odor identification) were added to the composite olfactory score. Results with low cores indicated worse performance than did high scores. For example, a lower score of the Sniffin Sticks test in the group of subjects with BSP than in the group of healthy controls indicated that the result of the group of subjects with idiopathic BSP was worse than the result of the healthy controls. A composite olfactory score of lower than 30 was considered as hyposmia (Haehner et al. 2009).

### Gustatory testing

The Taste Strips were used for the assessment of the composite taste scores (Mueller et al. 2003). Each taste strip was impregnated with one of the four flavors in four different concentrations. The four flavors were "sweet", "sour", "salty", or "bitter". There were also two strips without taste, the so-called “blanks”. The participants were presented the 18 strips in a predetermined order based on increasing flavor concentration, but the four flavors were presented in randomized order. Sufficient breaks were provided, and the participants were asked to drink some water regularly in between tastes, to avoid falsifying the results. In a forced-choice procedure, the participants were requested to indicate which taste they noticed. To evaluate the test, the number of correct answers was added (max. 16 points). The results of the two blanks were not recorded. A lower score in the Taste Stripe test indicated a worse performance than did a higher score. A composite taste score of lower than 9 was considered as hypogeusia (Mueller et al. 2003).

### Statistics

SPSS Statistics 25 software (SPSS Inc., Chicago, IL, USA) was used. The exact Mann–Whitney U test and Fisher’s test were used, since sample sizes were low and non-parametric tests are less likely to be affected by outliers. In the case of statistically significant differences between BSP subjects and healthy controls, multiple linear regression analysis was performed to assess whether clinical characteristics of BSP predicted performance on the tests of the chemical sense. Factors included were age, sex, education, disease duration of BSP, BoNT treatment, JRS, presence of tremor, saliva production, performance on the cognitive tests, as well as BSI depression and anxiety sub-scores. The critical *p* value was set to 0.05.

The study was approved by the local ethics committee (BB 004/19) and was performed in accordance with the ethical standards laid down in the 1964 Declaration of Helsinki and its later amendments. All study participants gave their informed consent before their inclusion in the study.

## Results

### Olfactory and gustatory ability in BSP subjects and healthy controls

**Fig. 1:**
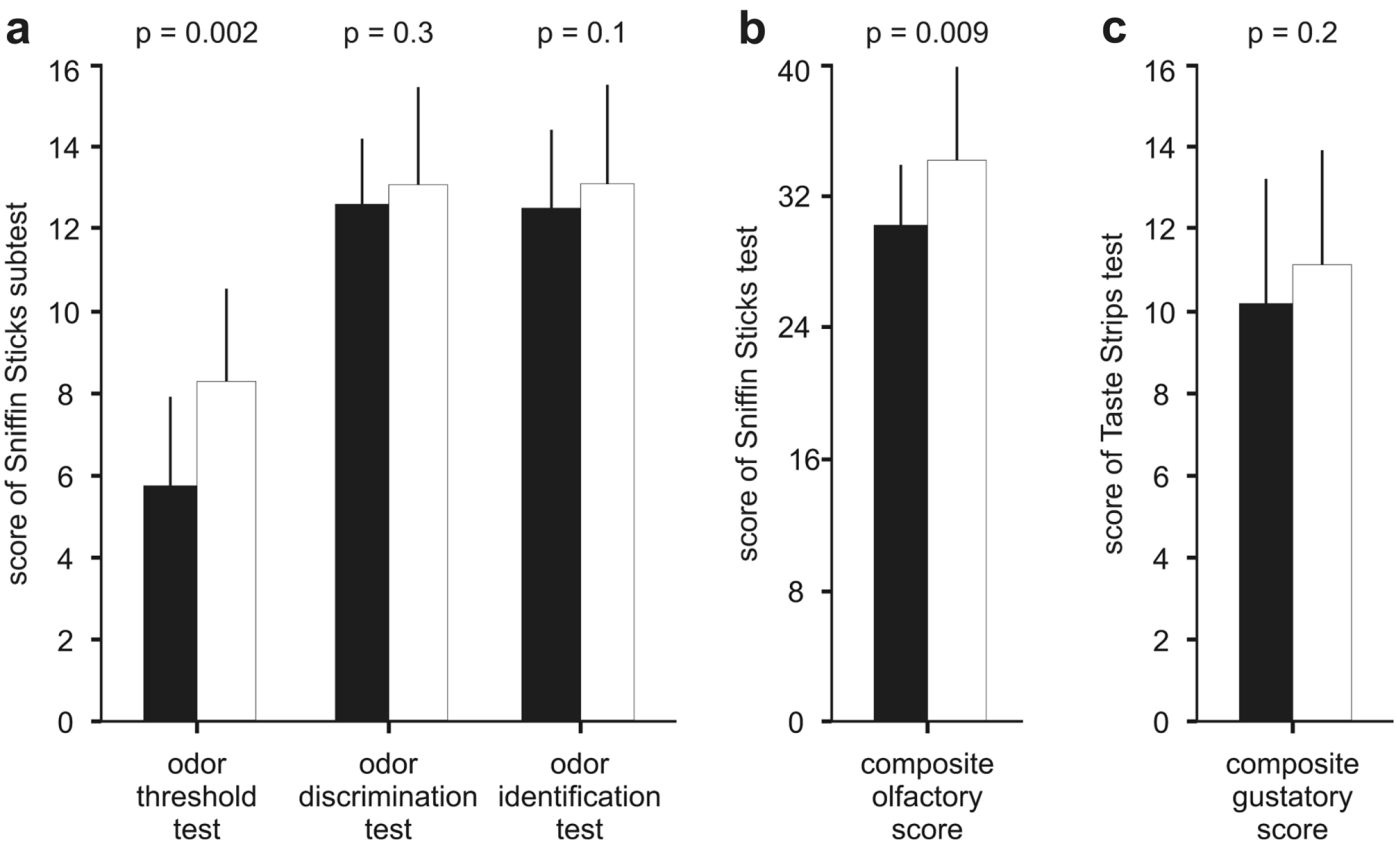
The figure shows the results of the three Sniffin Sticks sub-scores (**a**), results of the composite olfactory score from the Sniffin Sticks (**b**), and the results of the composite gustatory score from the Taste Strips (**c**). The black bars represent the results for the subjects with idiopathic blepharospasm and the white bars represent the results for the healthy controls. Values are presented as means and standard deviations. *P* values refer to the results of the Mann–Whitney U test

For this study, 115 subjects with BSP were screened. Details on the subjects with BSP screened and the number as well as causes of exclusion can be found in the flow chart in Fig. 1 in the Electronic Supplementary Material. Also, 26 healthy controls were screened (please see flowchart in Fig. 2 in the Electronic Supplementary Material). Seventeen subjects with idiopathic BSP and 17 healthy controls, who were willing to voluntarily participate, met the inclusion criteria, and did not have any exclusion criteria as defined by the study protocol, were finally included in this study as study participants. Additionally, healthy controls were matched for age, sex, education, and handedness of the subjects with idiopathic BSP. Population characteristics of the study participants are summarized in Table 1. All study participants were right-handed and Caucasian. Two subjects with idiopathic BSP had a slight action tremor of the hands consistent with tremor associated with dystonia (Bhatia et al. 2018).

**Table 1:** Demographic and clinical data of the study participants

	BSP subjects	Healthy controls	*p* value
Number of subjects	17	17	
Age, years	66.6 ± 8.4	66.7 ± 8.3	0.9
Sex (female/male/intersex)	12/5/0	12/5/0	1*
Education, years	9.8 ± 1.7	10.2 ± 1.9	0.6
ΔWeight cotton swabs, g	1.9 ± 0.9	1.8 ± 1.4	0.8
MoCA	29.0 ± 1.4	29.1 ± 1.1	0.7
ΔTMT, s	49.2 ± 22.7	56.8 ± 30.7	0.4
DST	16.3 ± 4.2	16.6 ± 2.8	0.5
FAS	37.6 ± 8.9	43.2 ± 13.5	0.2
BSI, depression sub-score	2.2 ± 4.5	1.4 ± 1.5	0.6
BSI, anxiety sub-score	2.6 ± 2.6	1.9 ± 2.1	0.5
Disease duration of BSP, years	9.8 ± 7.0		
BSRS total	8.3 ± 2.7		
JRS, severity	2.3 ± 0.8		
JRS, frequency	2.9 ± 0.7		

Values are mean ± standard deviation; BSP, blepharospasm; *p* values marked with *are based on the Fisher test; otherwise, exact Mann–Whitney U test was applied; MoCA, Montreal Cognitive Assessment (Freitas et al. 2012); ΔTMT, Difference between Trail-Making-Test part B—part A (Brown et al. 1958); DST, sum of Digit-Span-Test (de Paula et al. 2016); FAS, FAS-Test (Machado et al. 2009); BSI, Brief symptom inventory (Franke 2000); BSRS, Blepharospasm Severity Rating Scale (Defazio et al. 2015); JRS, Jankovic Rating Scale (Jankovic and Orman 1987).

The group of BSP subjects had a lower (= worse) odor threshold (5.7 ± 2.1 versus 8.4 ± 2.2, *p* = 0.002) and a lower (= worse) composite olfactory score (30.6 ± 3.9 versus 34.6 ± 6.0, *p* = 0.009) than the group of healthy controls, while odor discrimination (12.5 ± 1.6 versus 13.1 ± 2.3, *p* = 0.3) and odor identification (12.4 ± 1.9 versus 13.1 ± 2.5, *p* = 0.1) were not statistically different between the two groups (Fig. 1a,b). There were 8 subjects with idiopathic BSP (that is, 47% of the group of subjects with idiopathic BSP studied) and 3 healthy controls (that is, 18% of the group of healthy controls studied) with hyposmia. The difference in the prevalence of hyposmia in the two groups compared was only marginally significant (*p* = 0.06) as assessed through the Fisher’s test. An odor threshold below the 10th percentile of the norm (that is, a score below 5.75 points) is regarded as a pathological odor threshold (Oleszkiewicz et al. 2019). Based on this criterion, 8 out of 17 subjects with idiopathic BSP had a pathologic odor threshold, which represents 47% of the group of subjects with idiopathic BSP studied. In comparison, only one healthy control had a pathologic odor threshold, which represents 6% of the group of healthy controls studied. The comparison of subjects with a pathologic odor threshold in each group through the use of the Fisher test revealed that this difference was statistically significant (*p* = 0.017). Subjective olfactory functioning in subjects with idiopathic BSP was lower (= worse) than in healthy controls, but the difference was not statistically significant (7.0 ± 1.7 versus 7.8 ± 1.6, *p* > 0.05). The subjects with idiopathic BSP were not aware of their olfactory dysfunction. None of the subjects with idiopathic BSP described that they had an olfactory impairment as indicated by a subjective olfactory ability rating of lower than 5. Additionally, there were non-significant poor correlations between subjective olfactory functioning and results of the Sniffin Sticks test in the subjects with idiopathic BSP (Spearmen correlation coefficient between 0.2 to − 0.2; *p* values > 0.3). Multiple linear regression analysis revealed that neither demographic characteristics, motor manifestations as assessed with the JRS nor non-motor alterations of BSP including performance with the cognitive tests as well as the BSI were predictors for low odor threshold or the composite olfactory score in subjects with idiopathic BSP.

The group of subjects with idiopathic BSP had a slightly lower (= worse) composite gustatory score than the group of healthy controls (10.3 ± 3.1 versus 11.7 ± 2.9, *p* = 0.2) (Fig. 1c), and more subjects with idiopathic BSP had hypogeusia than healthy controls (29% versus 6%, *p* > 0.05), but the differences were statistically non-significant. Subjective gustatory functioning in subjects with idiopathic BSP was not found to differ compared to healthy controls (7.4 ± 1.7 versus 7.9 ± 1.5, *p* > 0.05).

### Effects of BoNT treatment on the chemical senses in subjects with BSP

In an adjacent study, 8 subjects with idiopathic BSP on BoNT treatment in standard doses (2 treated with abobotulinumtoxin A, 2 treated with onabotulinumtoxin A, and 4 treated with incobotulinumtoxin) were assessed twice. Details on the BoNT doses used in the subjects with idiopathic BSP in this study can be found in Electronic Supplementary Material Table 1. Four BSP subjects were assessed before the BoNT injections, and 4 weeks after, they had obtained their BoNT injections as part of routine care, while the other 4 subjects with idiopathic BSP on BoNT treatment were assessed in reverse order. For comparison, eight healthy controls were assessed twice, 4 weeks apart. The statistical analyses revealed that BoNT treatment had no effect on the chemical senses of the subjects with idiopathic BSP studied (Table 2).

**Table 2:** Effects of botulinum toxin on olfactory and gustatory functioning in subjects with blepharospasm compared to healthy controls

	BSP subjects		Healthy controls		*p* value
	Before BoNT	With BoNT	1. exam	2. Exam	
Number of subjects	8		8		
Age, years	69.7 ± 5.4		66.6 ± 7.3		
Sex (female/male/intersex)	5/3/0		6/2/0		
Education, years	10.1 ± 2.0		10.2 ± 1.9		
Disease duration, years	10.6 ± 8.3		–		
ΔWEIGHT cotton swabs, g	2.0 ± 1.2	2.2 ± 1.5	1.7 ± 1.4	1.5 ± 1.1	0.4
Composite olfactory score	32.6 ± 3.4	32.5 ± 3.9	35.9 ± 4.8	35.7 ± 3.7	0.9
Odor threshold	6.5 ± 2.1	6.8 ± 1.9	8.1 ± 1.3	8.0 ± 1.4	0.2
Odor discrimination	12.9 ± 1.6	12.4 ± 1.9	13.8 ± 1.9	13.6 ± 1.4	0.5
Odor identification	13.3 ± 1.2	13.3 ± 1.5	14.0 ± 2.1	14.1 ± 1.6	0.7
Composite gustatory score	10.9 ± 2.7	11.4 ± 2.9	11.1 ± 2.9	12.9 ± 1.6	0.3
JRS, severity	2.3 ± 0.5	3.5 ± 1.5			
JRS, frequency	2.9 ± 0.8	3.5 ± 1.5			
Global assessment		+ 2.3 ± 0.9			

Values are mean ± standard deviation; BSP, blepharospasm; BoNT, botulinum toxin; before BoNT, assessment just before the next BoNT injections; after BoNT, assessment 4 weeks after BoNT injections; p value as assessed with exact Mann–Whitney U test to compare the difference between before BoNT injections and 4 weeks thereafter in the BSP subjects with the difference between the first and the second assessment of the healthy controls; ΔWeight cotton swabs, difference of weight of two cotton swabs before and after the study participants kept them in their check pouch for 1 min. JRS, Jankovic Rating Scale (Jankovic and Orman 1987); Global Assessment, self-assessment of the effect of BoNT on BSP by the BSP subjects comparing the status 4 weeks after the last BoNT injections with the status before the BoNT injections (Defazio et al. 2015)

## Discussion

To contribute to the better understanding of idiopathic BSP, the primary aim of this study was to systematically analyze the sense of smell and the sense of taste in idiopathic BSP. Therefore, we assessed 17 subjects with idiopathic BSP compared to 17 healthy controls matched for age, sex, education, and handedness. The main findings are that the group of subjects with idiopathic BSP had a lower (= worse) odor threshold and a lower (= worse) composite olfactory score than the group of healthy controls, while the sense of taste was not impaired in subjects with idiopathic BSP compared to healthy controls. An adjacent study showed that BoNT did not affect the chemical senses in the subjects with idiopathic BSP studied.

Olfactory dysfunction can be classified according to the underlying etiology such as olfactory dysfunction secondary to sinonasal disease, post-infectious olfactory dysfunction, and post-traumatic olfactory dysfunction (Hummel et al. 2016). As we have excluded other potential causes of olfactory dysfunction via history and clinical exam, the olfactory decline we found in subjects with idiopathic BSP may be classified as olfactory dysfunction associated with neurological disease (Hummel et al. 2016). Through the use of a well-established test to assess different olfactory abilities, the group of subjects with idiopathic BSP was found to have a lower (= worse) odor threshold compared to the group of healthy controls. Additionally, more subjects with idiopathic BSP had a pathological odor threshold than did healthy controls. Findings from functional neuroimaging studies in healthy human subjects (Savic et al. 2000; Suzuki et al. 2001; Kareken et al. 2003; Kjelvik et al. 2012) and clinical studies in patients with degenerative or focal disease of the brain (Abele et al. 2003; Mainland et al. 2005; Kronenbuerger et al. 2010; Zobel et al. 2010) suggest that structures such as the cerebellum, the thalamus, and the sensorimotor cortex as well as their connecting circuits are involved in odor threshold (Sobel et al. 1998; Johnson et al. 2003; Mainland et al. 2005). Further research is needed to investigate which pathophysiological mechanisms may contribute to altered odor threshold in subjects with idiopathic BSP and whether disturbances exist within these structures and their connecting circuits.

The subjects with idiopathic BSP were not aware of their olfactory dysfunction. None of the subjects with idiopathic BSP described that they had an olfactory impairment as indicated by subjective ability rating of lower than 5 on a scale from zero (absent) to ten (superb). Additionally, there were non-significant poor correlations between the subjective olfactory rating and the results of the Sniffin Sticks test in the subjects with idiopathic BSP in this study. This is consistent with results of studies on the chemosensory system involving large numbers of humans where subjective olfactory rating poorly correlated with more objective olfactory tests such as the Sniffin Sticks test (Nordin et al. 1995; Delank and Stoll 1998; Landis et al. 2003; Doty 2005; Wehling et al. 2011; Knaapila et al. 2017; Adams et al. 2017; Hummel et al. 2017; Lötsch and Hummel 2019). Thus, olfactory deficits may be present without that the subjects being aware of it (Landis et al. 2003; Adams et al. 2017; Hummel et al. 2017). Therefore, the use of more objective olfactory tests such as the Sniffin Sticks test is recommended when it comes to the assessment of olfactory abilities (Hummel et al. 2017). However, subjective olfactory ratings may be considered as part of a comprehensive test battery of olfaction (Hummel et al. 2017).

Using the same study set-up, our previous study found that CD subjects had diminished (= worse) odor threshold in addition to impairment of odor identification and impairment of gustatory ability (Herr et al. 2020). This stands in contrast to the findings of the present study, where subjects with idiopathic BSP were found only to have diminished (= worse) odor threshold and not diminishment in taste. Although the sensorimotor network is involved in the pathophysiology of focal dystonia, the degree of alteration within this network may differ (Jinnah and Hess 2018). For instance, findings from imaging studies suggest that the microstructure of cortical and subcortical brain regions in CD differs from BSP (Obermann et al. 2007; Berman et al. 2018). Further research may focus on the question if this difference may have possibly contributed to the contrasting findings in idiopathic BSP and CD in terms of the chemical senses.

The adjacent study revealed that BoNT treatment did not alter the chemical senses when it was used in standard doses to treat BSP. Yet, further study is needed to clarify whether BoNT is safe regarding its impact on the chemical senses in BSP. It may be of interest if treatment with higher doses of BoNT—*where medically indicated—*may impact the chemical senses. For instance, for the treatment of subjects with CD, higher doses of BoNT are used than are in subjects with BSP (Hallett et al. 2013; Hassell and Charles 2020). Thus, a future study may assess the impact of BoNT in the chemical senses in subjects with CD treated with BoNT.

This study has strengths and limitations. This is the first study of the chemical senses in idiopathic BSP and it is the first study that assessed the impact of BoNT on the sense of smell and taste. Additionally, the subjects with idiopathic BSP in this study were carefully selected and systematically examined. Furthermore, subjects with causes of olfactory or gustatory decline other than idiopathic BSP were excluded from this study. This led to a limited number of subjects with idiopathic BSP assessed. As a result, co-factors of olfactory decline in subjects with idiopathic BSP with small-to-moderate impact may have been missed. Future studies assessing a larger number of subjects with idiopathic BSP may confirm whether olfactory decline is a non-motor manifestation separate from other non-motor alterations of idiopathic BSP. Key symptoms of the novel coronavirus (COVID-19) include loss of smell and taste (Whitcroft and Hummel 2020). As this study was completed before the outbreak of COVID-19 in Germany, the olfactory dysfunction found in idiopathic BSP was most likely not caused by COVID-19. However, COVID-19 may further worsen olfactory functioning in subjects with idiopathic BSP. This may be the focus of a separate study.

In conclusion, in this study, subjects with idiopathic BSP had a lower (= worse) odor threshold than healthy controls. As olfaction is important in daily life, findings justify further study on olfactory abilities in a larger number of subjects with idiopathic BSP.

## Supplementary Information

Below is the link to the electronic supplementary material.

Supplementary file1 (PDF 91 KB)

Supplementary file2 (PDF 25 KB)

Supplementary file3 (PDF 22 KB)

## Data Availability

All authors had full access to all data collected and analyzed for this study. The data that support the findings of this study are available from the corresponding author upon reasonable request.

## Declarations

### Code of availability

SPSS Statistics 25 software (SPSS Inc., Chicago, IL, USA) was used.
