# Supplementary material for: Smell and taste in idiopathic blepharospasm
Source: J Neural Transm (Vienna). 2021 Jun 28;128(8):1215–24. doi: 10.1007/s00702-021-02366-4 (PMC8237775; doi:10.1007/s00702-021-02366-4)
Supplement: Supplementary file 1 — Supplementary file1 (PDF 91 KB) [file 702_2021_2366_MOESM1_ESM.pdf]

Article Titel: Smell and taste in idiopathic blepharospasm.
Journal Name: Journal of Neural Transmission
Author Names: Julie Gamain[1], Thorsten Herr[1], Robert Fleischmann MD[1], Andrea Stenner MD[2], Marcus Vollmer PhD[3], Carsten Willert MD[4], Birgitt Veit MD[5], Bernhard Lehnert MD[6], Jan-Uwe Mueller MD[7], Frank Steigerwald MD[1], Frank Tost MD[8], Martin Kronenbuerger MD[1,9,10]
Affiliations:
[1]Department of Neurology, University of Greifswald, Greifswald, Germany
[2]Department of Neurology, Paracelsus Clinic Zwickau, Zwickau, Germany
[3]Institute of Bioinformatics, University of Greifswald, Greifswald, Germany
[4]Neurology Group Practice, Stralsund, Germany
[5]Neurology Group Practice, Neubrandenburg, Germany
[6]Department of Otorhinolaryngology, University of Greifswald, Greifswald, Germany
[7]Department of Neurosurgery, University of Greifswald, Greifswald, Germany
[8]Department of Ophthalmology, University of Greifswald, Greifswald, Germany
[9]Department of Neurology, Johns Hopkins University, Baltimore, Maryland, USA
[10]Department of Neurology, Medical School OWL, University of Bielefeld, Germany
Email for corresponding author: (Martin Kronenbuerger) martinkro2@hotmail.com

Electronic Supplementary Material Table 1 Botulinum toxins used in subjects with blepharospasm

Subject, ID	Botulinum toxin used	dose per eye, U
1	Abobotulinum toxin A	140
2	-	-
3	Onabotulinum toxin A	81
4	Incobotulinum toxin A	40
5	Abobotulinum toxin A	152
6	-	-
7	Incobotulinum toxin A	60
8	Incobotulinum toxin A	30
9	Incobotulinum toxin A	30
10	-	-
11	-	-
12	Onabotulinum toxin A	48
13	-	-
14	Incobotulinum toxin A	112
15	Onabotulinum toxin A	49
16	Abobotulinum toxin A	200
17	Abobotulinum toxin A	200

ID, identification number; U, units; Subjects 1, 3, 4, 5, 7, 8, 9 and 12 participated in the adjacent study on the effects of botulinum toxin on the chemical senses; “-“, subjects with blepharospasm, who never had botulinum toxin or stopped botulinum toxin treatment more than 6 months before the study.
