# Supplementary material for: Smell and taste in idiopathic blepharospasm
Source: J Neural Transm (Vienna). 2021 Jun 28;128(8):1215–24. doi: 10.1007/s00702-021-02366-4 (PMC8237775; doi:10.1007/s00702-021-02366-4)
Supplement: Supplementary file 2 — Supplementary file2 (PDF 25 KB) [file 702_2021_2366_MOESM2_ESM.pdf]

Article Titel: Smell and taste in idiopathic blepharospasm.
Journal Name: Journal of Neural Transmission
Author Names: Julie Gamain[1], Thorsten Herr[1], Robert Fleischmann MD[1], Andrea Stenner MD[2], Marcus Vollmer PhD[3], Carsten Willert MD[4], Birgitt Veit MD[5], Bernhard Lehnert MD[6], Jan-Uwe Mueller MD[7], Frank Steigerwald MD[1], Frank Tost MD[8], Martin Kronenbuerger MD[1,9,10]
Affiliations:
[1]Department of Neurology, University of Greifswald, Greifswald, Germany
[2]Department of Neurology, Paracelsus Clinic Zwickau, Zwickau, Germany
[3]Institute of Bioinformatics, University of Greifswald, Greifswald, Germany
[4]Neurology Group Practice, Stralsund, Germany
[5]Neurology Group Practice, Neubrandenburg, Germany
[6]Department of Otorhinolaryngology, University of Greifswald, Greifswald, Germany
[7]Department of Neurosurgery, University of Greifswald, Greifswald, Germany
[8]Department of Ophthalmology, University of Greifswald, Greifswald, Germany
[9]Department of Neurology, Johns Hopkins University, Baltimore, Maryland, USA
[10]Department of Neurology, Medical School OWL, University of Bielefeld, Germany
Email for corresponding author: (Martin Kronenbuerger) martinkro2@hotmail.com

Electronic Supplementary Material Figure 1 Flow chat about screening of subjects with blepharospasm

Subjects who presented with blepharospasm between January 2015 and January 2020 to the Ophthalmology & Neurology Department outpatient clinics, University Hospital Greifswald (n = 115)

Excluded due to contraindications as defined per study protocol (n = 84)

- Use of medication know to impede the chemical senses (n = 14)
- Abnormal findings on routine neuroimaging studies (n = 3)
- Additional ophthalmological diseases (n = 10)
- Additional neurological diseases (n = 30)
- Abnormal laboratory findings (n = 7)
- History of head trauma (n = 2)
- Sinonasal disease (n = 8)
- Tobacco smoking (n = 10)

Subjects with blepharospasm, who met the inclusion criteria and did not have exclusion criteria based on the available information in the hospital electronic record system. Subjects were mailed a letter about the study and invited to contact the investigators if they were interested to participate (n = 31).

Subjects with blepharospasm invited to the study and who responded to the invitation letter. Subjects were interviewed over the phone if they met the inclusion and did not have exclusion criteria (n = 24).

Excluded due to contraindications as defined per study protocol (n = 7)

- Spread of dystonia to jaw and cervical muscles (n = 2)
- Started to smoke tobacco recently (n = 2)
- Repeated no-show (n = 1)
- Sinonasal disease (n = 2)

Subjects presented to the study appointment, examined and interviewed in-person if they met the inclusion and did not have exclusion criteria. Subjects with idiopathic blepharospasm included (n = 17).
