# Supplementary material for: Smell and taste in idiopathic blepharospasm
Source: J Neural Transm (Vienna). 2021 Jun 28;128(8):1215–24. doi: 10.1007/s00702-021-02366-4 (PMC8237775; doi:10.1007/s00702-021-02366-4)
Supplement: Supplementary file 3 — Supplementary file3 (PDF 22 KB) [file 702_2021_2366_MOESM3_ESM.pdf]

Article Titel: Smell and taste in idiopathic blepharospasm.
Journal Name: Journal of Neural Transmission
Author Names: Julie Gamain[1], Thorsten Herr[1], Robert Fleischmann MD[1], Andrea Stenner MD[2], Marcus Vollmer PhD[3], Carsten Willert MD[4], Birgitt Veit MD[5], Bernhard Lehnert MD[6], Jan-Uwe Mueller MD[7], Frank Steigerwald MD[1], Frank Tost MD[8], Martin Kronenbuerger MD[1,9,10]
Affiliations:
[1]Department of Neurology, University of Greifswald, Greifswald, Germany
[2]Department of Neurology, Paracelsus Clinic Zwickau, Zwickau, Germany
[3]Institute of Bioinformatics, University of Greifswald, Greifswald, Germany
[4]Neurology Group Practice, Stralsund, Germany
[5]Neurology Group Practice, Neubrandenburg, Germany
[6]Department of Otorhinolaryngology, University of Greifswald, Greifswald, Germany
[7]Department of Neurosurgery, University of Greifswald, Greifswald, Germany
[8]Department of Ophthalmology, University of Greifswald, Greifswald, Germany
[9]Department of Neurology, Johns Hopkins University, Baltimore, Maryland, USA
[10]Department of Neurology, Medical School OWL, University of Bielefeld, Germany
Email for corresponding author: (Martin Kronenbuerger) martinkro2@hotmail.com

Electronic Supplementary Material Figure 2 Flow chat about screening of healthy controls

Flyers about the study distributed at the Ophthalmology and Neurology Department outpatient clinics at the University Hospital Greifswald.

↓

Healthy controls, who responded to the flyer and contracted the investigators. Healthy controls were interviewed over the phone if they met the inclusion criteria and did not have exclusion criteria (n = 26).

→ Excluded due to contraindications as defined per study protocol (n = 9)

- Did not match to age, sex, education, and handedness of BSP subjects (n = 5)
- Use of medication know to impede the chemical senses (n = 2)
- Sinonasal disease (n = 2)

↓

Healthy controls, who presented to the study appointment were examined and interviewed in-person if they met the inclusion criteria and did not have exclusion criteria. Healthy controls included (n = 17).
